# Intravitreal Administration of a Selective HDAC6 Inhibitor Prevents Retinal Damage Progression in the Acute Ocular Toxoplasmosis Model

**DOI:** 10.1021/acsinfecdis.5c00818

**Published:** 2026-01-14

**Authors:** Carlla Assis Araujo-Silva, Milena Ribeiro Peclat-Araujo, Vanderlei da Silva Fraga-Junior, Thuany Prado-Rangel, Dio Pablo Alexandrino-Mattos, Claudia Farias Benjamim, Christina Maeda Takiya, Wanderley de Souza, Rossiane Claudia Vommaro

**Affiliations:** † Laboratório de Ultraestrutura Celular Hertha Meyer, Centro de Pesquisa em Medicina de Precisão, Instituto de Biofísica Carlos Chagas Filho, 28125 Universidade Federal do Rio de Janeiro, Cidade Universitária, Rio de Janeiro, RJ 21941-599, Brasil; ‡ Instituto Nacional de Ciência e Tecnologia em Biologia Estrutural e Bioimagens, Universidade Federal do Rio de Janeiro, Rio de Janeiro 21941-630, Brasil; § Laboratório de Imunologia Molecular e Celular, Instituto de Biofísica Carlos Chagas Filho, 204227 Universidade Federal do Rio de Janeiro, Cidade Universitária, Rio de Janeiro, RJ 21941-904, Brasil; ∥ Laboratório de Imunopatologia, Instituto de Biofísica Carlos Chagas Filho, Universidade Federal do Rio de Janeiro, Cidade Universitária, Rio de Janeiro, RJ 21941-902, Brasil; ⊥ Laboratório de Terapia Gênica e Vetores Virais, Centro de Pesquisa Em Medicina de Precisão, Instituto de Biofísica Carlos Chagas Filho, Universidade Federal do Rio de Janeiro, Cidade Universitária, Rio de Janeiro, RJ 21941-599, Brasil

**Keywords:** retina pathology, ocular disease, immunomodulation, experimental chemotherapy, tubastatin A

## Abstract

Ocular toxoplasmosis (OT), caused by *Toxoplasma gondii*, is the leading cause of retinochoroiditis worldwide, with particularly severe cases in Brazil. The treatment used for OT is the combination of cotrimoxazole and corticosteroids. However, this therapy includes prolonged treatment, resistance to circulating strains, and cytotoxic effects for patients. The intensification of the inflammatory response against *T. gondii* can exacerbate retinal tissue damage. In this study, the HDAC6 inhibitor Tubastatin A was evaluated by intravitreal injection in the murine ocular toxoplasmosis model. Tubastatin A has presented anti-*T. gondii* activity and an interesting potential for immunoregulation in the approach to eye disease. The inhibition of HDAC6 interferes with the establishment of infection by blocking the recruitment of the host cell cytoskeleton, which is necessary for the active entry of tachyzoites. After 5 days of treatment, Tubastatin A prevented the progression of lesions in the infected retina from the 10th postinfection day. Tubastatin A restored retinal tissue barriers and regulated the HDAC6-Hsp90 pathway, leading to decreased VEGF and HSF1 expression, which may help prevent neovascularization observed in OT patients. A single intravitreal dose of Tubastatin A established an anti-inflammatory microenvironment that supported retinal tissue homeostasis. Tubastatin regulated micro- and macroglial activation, reduced immunolabeling of Iba1 and GFAP (glial fibrillary acidic), and decreased the secretion of IL-12, IL-4, and IL-17A, key cytokines associated with OT pathology. The combination of Tubastatin A with antifolates may be a viable new treatment regimen to protect retinal tissue and prevent blindness in patients.

## Graphical abstract

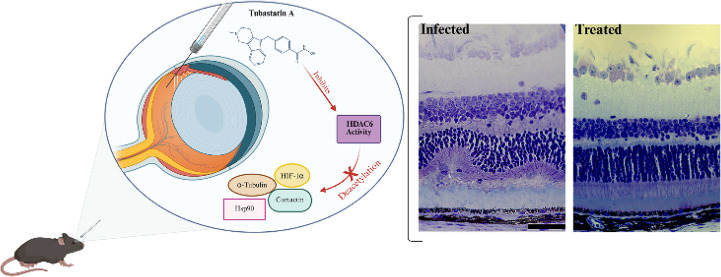

Ocular toxoplasmosis (OT) is a clinically relevant manifestation of acute infection with *Toxoplasma gondii*. OT has been identified as a primary cause of infectious posterior uveitis, with a predominance in North, Central, and South America, and in several African countries.^1,2^ OT can lead to retinochoroiditis, resulting in substantial retinal damage and potentially leading to blindness.^3,4^

The standard chemotherapy regimen for OT is a combination of pyrimethamine and sulfadiazine or cotrimoxazole (trimethoprim-sulfamethoxazole), which exhibit a synergistic effect by inhibiting enzymes involved in folic acid synthesis.^5^ Furthermore, the association of oral corticosteroids is recommended due to an intense active inflammatory response in retinal tissue. An alternative treatment utilized in clinical practice involves the intravitreal injection of clindamycin and corticosteroids.^4,6^ However, the study by Feliciano-Alfonso and collaborators concluded that alternative regimens do not achieve efficacy levels superior to those of the standard treatment.^7^

The tissue damage caused by focal lesions is not attributed solely to the proliferation of *T. gondii*, but to the inflammatory reaction generated in loco.^8,9^ The molecular mechanisms that regulate the Th1 inflammatory response in the ocular environment for *T. gondii* differ from those observed in other tissues.^10,11^ Although the retina presents a wide range of constitutively pro-inflammatory factors involved in eradicating pathogens and induces the upregulation of IL-12, IFN-γ, and TNF-α expression,^12,13^ the immunosuppressive aspect of the environment blocks the production of IFN-γ through anti-inflammatory mediators such as α-MSH (α-melanocyte-stimulating hormone), released by the retinal pigment epithelium (RPE), thrombospondin 1 (TSP1), programmed death ligand 1 (PDL1), TGF-β, Vasoactive Intestinal Peptide (VIP), and IL-10 to prevent irreversible damage.^10,14–17^ However, this immunoregulation can interfere with the control of parasite replication.

Given the immune-privileged status of the eye, retinal vasculature is enveloped by specialized structural barriers—the inner blood-retinal barrier (iBRB) and the outer blood-retinal barrier (oBRB)—which differ in both anatomical location and molecular composition. The components of these barriers are modulated by *T. gondii* infection, but the active inflammatory responses may also influence them. According to Lahmar and colleagues (2014),^18^ the iBHR response to *T. gondii* infection showed reduced expression of glial cell-specific proteins, such as GFAP, in astrocytes and Müller cells. The RPE constitutes the outer oBRB with the Bruch’s membrane (BM) and is in contact with the photoreceptor outer segments (OS).^19^ In vitro investigations with RPE cells cocultured with neutrophils and infected with tachyzoites showed increased ROS secretion, as well as the production of GM-CSF, IL-6, and IL-18.^20^ The THP-1 cell line (human monocytes), preinfected and cocultured with RPE cell line ARPE-19, exhibited decreased function of the junction proteins ZO-1 and occludin, and of the transepithelial electrical resistance (TEER).^21^ Moreover, studies of the pathophysiology of OT have identified alterations in the expression of pivotal factors, as the downregulation of the pigment epithelium-derived factor (PEDF) and overexpression of vascular endothelial growth factor A (VEGF-A).^22,23^ Clinical trials have demonstrated the potential of intravitreal therapy combining anti-VEGF agents with antiproliferative drugs for *T. gondii* to treat ocular neovascularization secondary to OT effectively.^24^

The repurposing of drugs is a widely recognized practice for developing new therapies.^25,26^ HDAC6 inhibitors have been explored in biological systems for several pathological conditions. Tubastatin A (TST) has been explored in neurodegenerative pathologies for its ability to modulate the cytoskeleton, stabilizing microtubules through α-tubulin and regulating cortactin activity.^27–29^ The protective action of this inhibitor also occurs through other target proteins, such as the fine regulation of chaperone complexes and their client proteins, such as Hsp90 and Hsp70.^28,30^ The HDAC genes in *T. gondii* already annotated in its genome have been explored in chemotherapy studies. The HDAC family, comprising the class I HDAC 2 and 3; the class II HDAC 1 and 5, and the class III deacetylases (Sirtuins) Sir2A and Sir2B have already been associated with the encystment process, proliferation, and maintenance of metabolic pathways; and TgHDAC4, a class IV HDAC, which has been found in the apicoplast.^31,32^ In vitro studies have shown the selective activity of TST against *T. gondii* tachyzoites, with an IC_50_ in the nanomolar range, resulting in a direct effect on the parasite endodyogeny process.^33^ The data from these experiments support the view that this inhibitor is promising for in vivo tests, and its immunomodulatory characteristics may be of interest in the OT context. Furthermore, previous studies demonstrated that the compound has potential as a therapeutic antiparasitic agent.^34^

In this study, we evaluated the hydroxamic acid derivative TST, a selective HDAC6 inhibitor, as an alternative intravitreal treatment for the murine model of acute ocular toxoplasmosis, given its protective effects in other nervous system models and its antiproliferative activity against *T. gondii* in vitro.^27–29,33^

## Results

### TST Protected the Tight and Adherent Junctions of the Retinal Pigment Epithelium

To confirm whether TST protects the integrity of RPE during *T. gondii* infection, ARPE-19 monolayers were infected with tachyzoites and treated with 5 μM TST for 1 or 2 h. Immunofluorescence microscopy and Western blotting were performed to analyze the expression of occluding and adherent junction proteins. Immunofluorescence revealed a 40% reduction in the zona occludent protein (ZO-1) staining induced by infection, whereas with TST treatment, this did not occur (Figure 1A–B). However, Western blot analysis showed no significant difference in ZO-1 expression levels among groups (Figure 1C). In this case, the modulation of ZO-1 is likely linked to its localization, rather than its expression level. Regarding adherent junctions, a critical effect was observed after 2 h of infection, with a 40% reduction in pan-cadherin expression. In the infected and treated group for 2 h, TST prevented the decline in the adherens junction expression caused by infection (Figure 1D). These findings suggest that TST prevented the reduction of tight and adherens junctions induced by infection, thereby helping to maintain the integrity of the RPE. To verify whether TST has a toxic effect on ARPE-19, cells were treated with different concentrations for 72 h. Cell viability was evaluated using the MTS assay. TST did not cause significant toxicity to ARPE-19 cells, with CC_30_ and CC_50_ values of 263 μM and 332 μM, respectively (Supporting Information Figure 1A and B).

**Figure 1:**
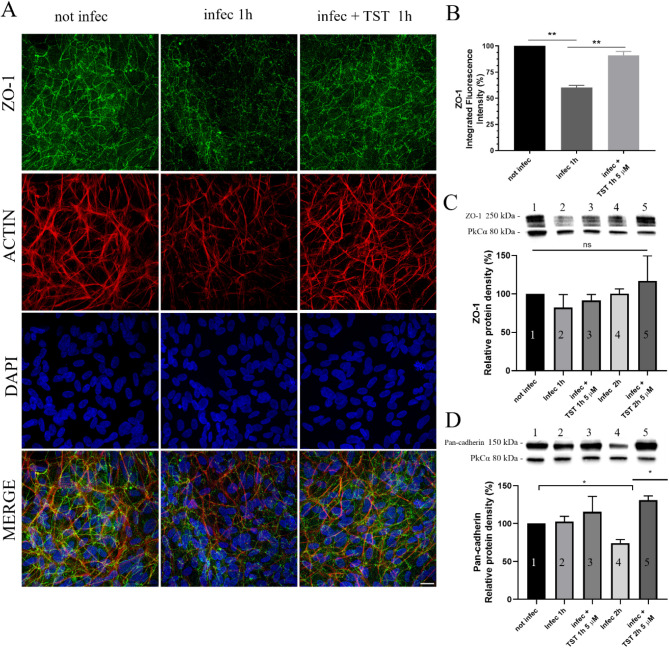
Confluent cultures of ARPE-19 infected with ME49 tachyzoites present junction proteins protected when treated with TST. (A) The protective effect is evidenced by the recovery of ZO-1 staining after infection with ME49 tachyzoites and treatment with 5 μM TST. Scale bar, 50 μm. (B) Quantification of integrated fluorescence intensity of ARPE-19, **P = 0.0034. (C–D) Relative amount of total protein using Western blotting. (C) ZO-1 protein did not change its amount after infection. (D) Cadherin class proteins decrease in quantity in cells infected with tachyzoites for 2 h. *P = 0.0434. Statistical significance was assessed using Tukey’s multiple comparisons test.

### The Invasion of Tachyzoites In Vitro Was Blocked after TST Treatment

To evaluate whether the HDAC6 inhibitor would block the entry of *T. gondii* and the establishment of infection, ARPE-19 cells were pretreated with 5 μM TST for 48 h before interaction with tachyzoites for 1 or 2 h. Immunofluorescence microscopy was used to analyze the recruitment of the host cell cytoskeleton, using phalloidin-rhodamine to probe for actin filaments and α-acetylated α-tubulin to localize acetylated microtubules. The immunofluorescence analysis showed that in the absence of TST, the internalization of the parasites was successful after 1 or 2 h, and the recruitment of the cytoskeleton was evident in the merged image of both stains around the parasitophorous vacuoles (PV) (Figure 2A,C—inset, orthogonal/3D render). In contrast, pretreatment with TST impaired the recruitment of these structures and the parasite’s entry (Figure 2B,D—inset, orthogonal/3D render). Scanning electron microscopy was also used to assess the parasite’s presence on the host cell surface (Supporting Information Figure 2). These results suggest that the TST treatment prevents the modulation of the host cell cytoskeleton necessary for the active ingress of tachyzoites, thereby interfering with the establishment of infection.

**Figure 2:**
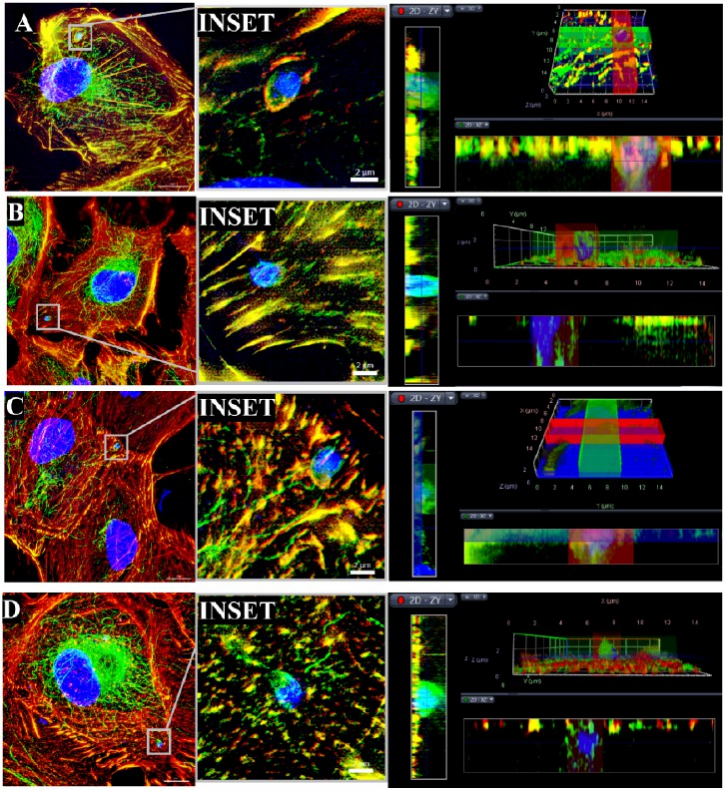
Invasion assay in ARPE-19 cells pretreated with TST for 48 h shows protection against tachyzoite invasion for 1 and 2 h of interaction. Analysis with Structured Illumination Microscopy (SIM) of maximum intensity projection (MIP) and 3D tomography showed that tachyzoites did not recruit actin filaments (546 nm) or microtubules (488 nm). (A) Cells were allowed to interact with tachyzoites for 1 h without pretreatment. (B) 1 h interaction with 48 h of TST pretreatment. (C) 2 h of interaction, without pretreatment. (D) 2 h of interaction, with pretreatment. Scale bars: 10 μm, Inset 2 μm.

### TST Protected RPE Homeostasis against *T. gondii* Infection In Vitro

To investigate changes in critical factors induced by infection in RPE that are important for maintaining intracellular parasite and host cell homeostasis, ARPE-19 cells were infected with tachyzoites and treated with 5 μM TST for 24 h. The mRNA analysis was performed using primers for *VEGFA*, thrombospondin-1 (*THBS1*), heat shock factor 1 (*HSF1*), and hypoxia-inducible factor 1-alpha (*HIF-1α*). This analysis showed that *T. gondii* infection increased *VEGFA* expression 100-fold and *HSF1* expression 52-fold after 24 h (Figure 3A,D). TST treatment significantly reduced the increase in *VEGFA* and *HSF1*, bringing levels back to near baseline levels. However, *THBS-1* expression declined in infected cells, and TST did not reverse this decline (Figure 3C). The findings indicated that TST can modulate the stress response to *T. gondii* infection by regulating angiogenesis factors and heat shock protein expression, thereby helping to maintain RPE homeostasis.

**Figure 3:**
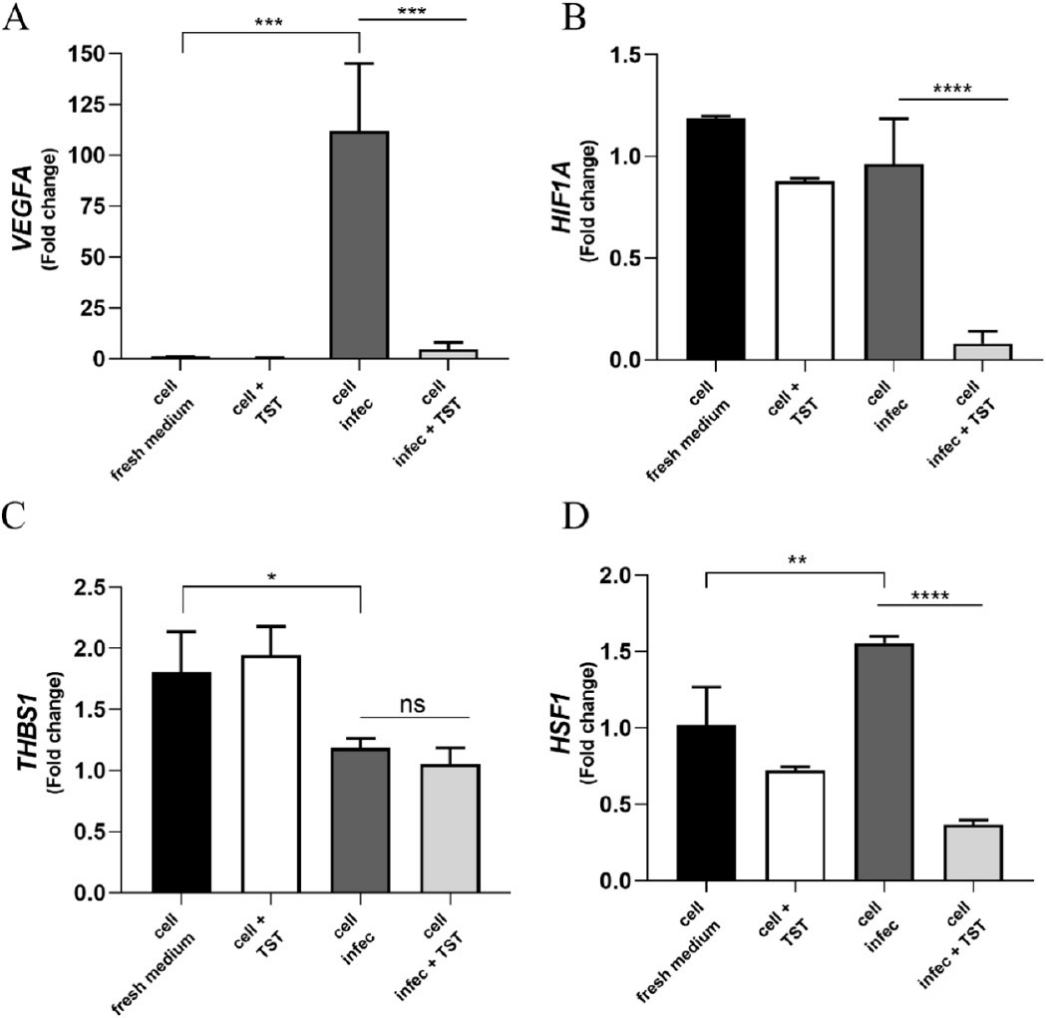
*T. gondii* infection modulates the expression of critical factors in RPE cells, influencing both homeostasis and cellular immune response. (A) The expression of the angiogenesis factor *VEGFA* was significantly increased in cells infected for 24 h; 24 h of treatment with TST prevented this regulation, ***P = 0.0003. (B) *HIF-1α* is involved in the regulation of *VEGFA* transcription, but its transcription remains unaltered by infection; treatment significantly decreased it compared to basal levels, ****P < 0.0001. (C) *THBS1* undergoes transcriptional alteration, and treatment did not reverse this decrease, *P = 0.0168. (D), **P = 0.0054, ****P < 0.0001. Statistical significance was assessed using Tukey’s multiple comparisons test.

### Intravitreal Injection of TST Was Safe In Vivo Model

To assess the potential toxic effects of TST on retinal tissue, male C57BL/6 mice (8–12 weeks old) were intravitreally treated with 10 μg/μL of TST in 2 μL per eye (Supporting Information Figure 4A). Histological analysis was performed 5 days postinjection (Figure 4). Control eyes from noninfected and nontreated animals were used for comparison (Figure 4A,B). The TST-injected eyes showed no changes in retinal layer architecture compared to the control eyes (Figure 4C,D). The results confirm that TST is a safe compound for intravitreal chemotherapy.

**Figure 4:**
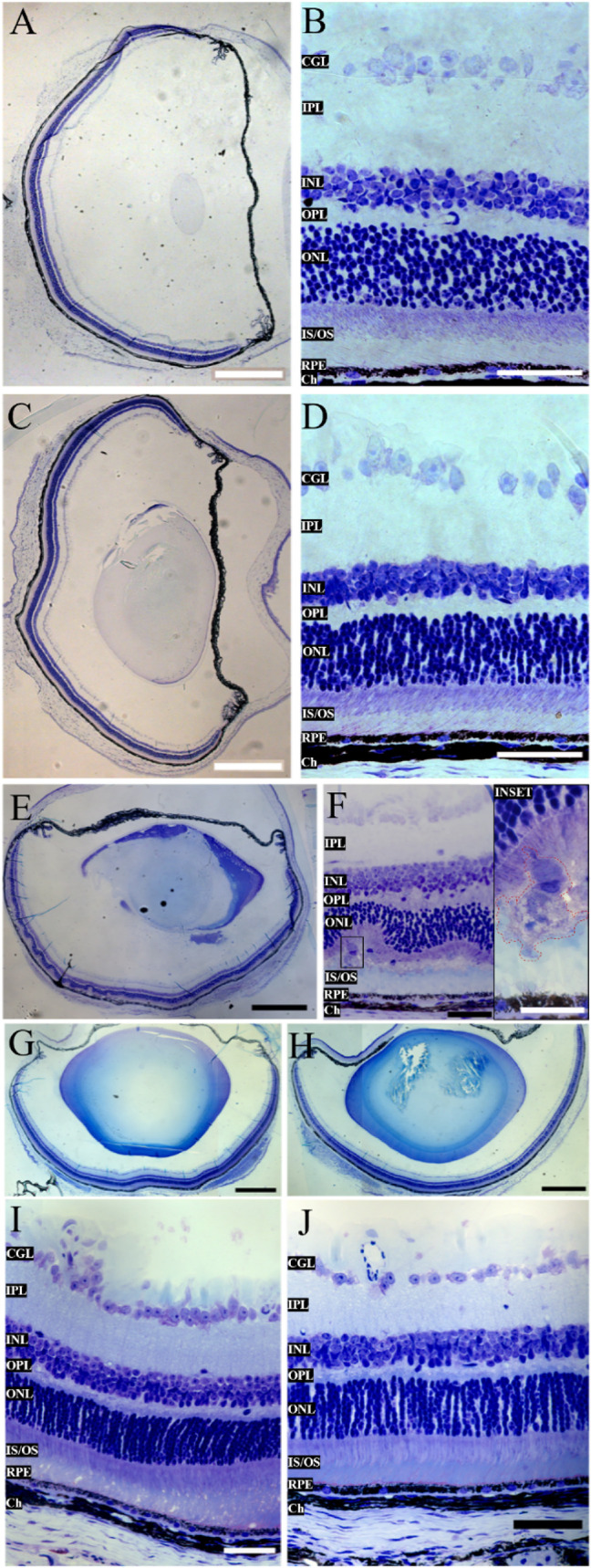
C57BL/6 animals with acute lesions caused by *T. gondii* showed improvement in their condition when treated with intravitreal TST. (A–B) Animals without infection. (C–D) Animals without infection with intravitreal injection of 20 μg/2 μL of TST did not show morphological alterations. (E–F) Animals with 18 days of infection, alteration of the layers, with cell infiltration (red dashed). (G, I) Right eye, infected, and treated. (H, J) Left eye, infected, and treated. Treated animals showed preserved structure of the IS/OS and ONL layers and integrity of the RPE. CGL: ganglion cells, IPL: inner plexiform layer, INL: inner nuclear layer, OPL: outer plexiform layer, ONL: outer nuclear layer, IS/OS: inner and outer segments of the photoreceptor, RPE: retinal pigment epithelium, Ch: choroid. Scale bars: A, C, E, G, H, 50 mm, and B, D, F, I, J, 50 μm.

### Intravitreal Injection of TST Avoided Conversion of *T. gondii* to the Bradyzoite Stage

To investigate whether TST could be indicated for drug repositioning to ocular toxoplasmosis (OT), male C57BL/6 mice (8–12 weeks old) were infected with 10^4^ tachyzoites of the ME49-GFP strain via intraperitoneal injection (i.p), as described in the OT model established in a previous study.^35^ On the 10th postinfection day, mice received a single dose of 10 μg/μL TST, with 2 μL administered per eye. After 5 days of treatment, the mice were euthanized for further analyses (the timeline of intravitreal treatment administration used in all experiments is shown in Supporting Information Figure 4B). Here, parasite load was analyzed by qPCR using the B1 gene marker of *T. gondii*. Stage-specific markers SAG1 (tachyzoites) and BAG1 (bradyzoites) were analyzed through mRNA expression using the RT-qPCR technique. The analysis showed that the load parasite was higher on the 10th postinfection day, which decreased on the 15th postinfection day without intervention. After 5 days of TST treatment, the parasite load remained unchanged compared with the untreated group at 15 days postinfection (Supporting Information Figure 3). Concerning the stage-specific markers, no significant difference in SAG1 levels was observed among groups. BAG1 levels showed a 1.5-fold increase in the 15-day postinfection group compared with the 10-day postinfection group (Supporting Information Figure 3). In the treated group, BAG1 levels were reduced by 3-fold compared to the nontreated group. These findings indicated that a single intravitreal dose of TST over a short period was not sufficient to reduce the parasite load; however, it interfered with the conversion of tachyzoites to bradyzoites. Therefore, further research with long-term and different concentrations is necessary to evaluate anti-*T. gondii* activity in vivo OT model.

### Intravitreal Injection of TST Prevented Retinal Damage Caused by *T. gondii* Infection

The effectiveness of TST in preventing the progression of retinal lesions caused by *T. gondii* infection was investigated. The assay was carried out as in the OT established model, as previously mentioned in item 2.6. In our previous studies, retinochoroiditis was observed in infected mice on the 15th day postinfection, progressing through the 20th day. It was associated with inflammatory cell infiltration and disruption of retinal tissue architecture. Mice were treated with a single intravitreal injection of TST on the 15th day postinfection, as described above, and euthanized on the 18th postinfection day. Histopathological analysis was conducted to evaluate morphological changes in the retinal tissue (Figure 4E–J). After 18 days postinfection, nontreated infected mice exhibited significant morphological alterations in retinal tissue, including the formation of photoreceptor rosettes due to detachment of these cells’ segments along with the RPE, and the presence of infiltrated cells in the segments of the inner (IS)/outer (OS) layers (Figure 4E,F). Regarding the treated infected mice, all 16 eyes exhibited preservation of the IS/OS region and the RPE. Infiltrated cells were absent in the retina (Figure 4G,J).

To investigate whether TST treatment could also be effective in the early stages of retinochoroiditis, another infected group was treated on the 10th postinfection day with a single injection of TST, as previously mentioned, and euthanized on the 15th postinfection day (Figure 5). After 5 days of treatment, the retinal tissue was analyzed, particularly in the IS/OS photoreceptor-RPE junctions and the RPE/Bruch’s membrane regions. Histopathological analyses of 1 μm sections showed IS/OS-RPE and RPE/Bruch’s membrane region. The respective regions were sectioned into 70 nm ultrathin slices and analyzed under a 100 kV microscope (Figure 5A,C,E,G) and (Figure 5B,D,F,H). The control mice that were not infected and were not treated (Figure 5A,B). As illustrated in Figure 5B, control mice showed that the interdigitations of the apical region of the RPE cells in contact with the OS were evident, and the structure of the basal lamina, or Bruch’s membrane, appeared well preserved. In infected mice, cell infiltrates were observed (Figure 5C, INSET, white arrow). In Figure 5D, the impact of the infection on the Bruch’s membrane can be seen; the general morphology was discontinuous in the inner collagenous layer (ICL) and outer collagenous layer (OCL), and presented an amorphous electron-dense debris (white bracket and white asterisk). After treatment, the same region illustrated in Figure 5F,H (white bracket) in infected mice exhibited preserved overall morphology, with the interdigitation area between the RPE and the basal lamina well maintained in both eyes. These results indicated that TST treatment could prevent the beginning of the lesion in the early days of infection or the worsening lesions when retinochoroiditis was established. However, it did not reduce the parasite load.

**Figure 5:**
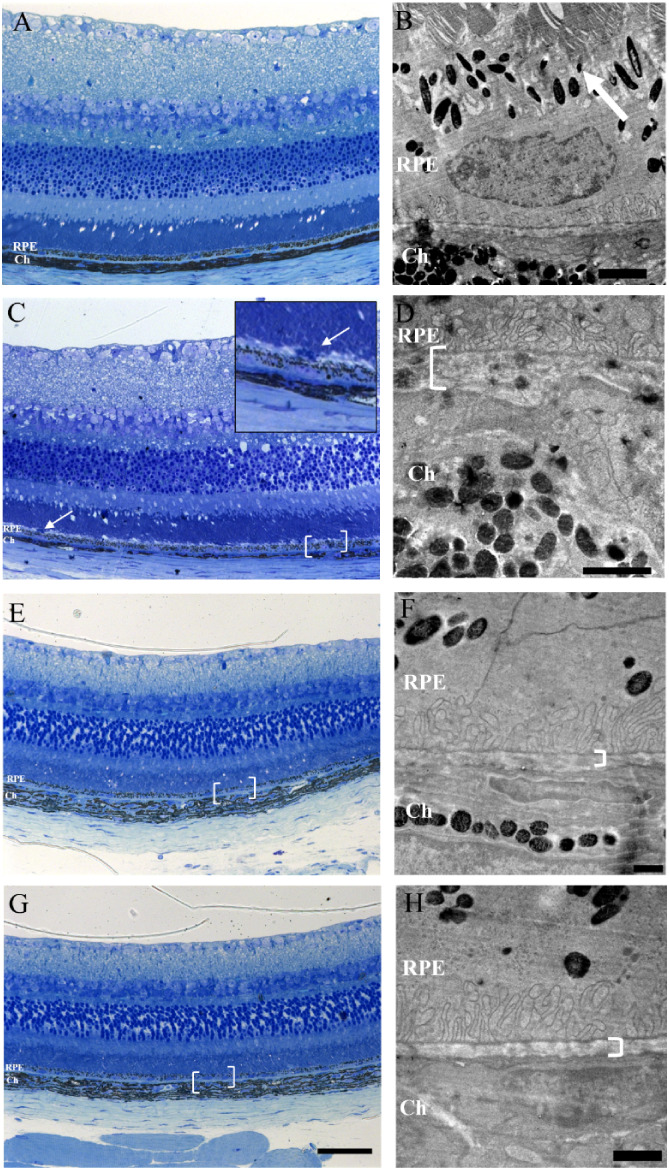
Light and transmission electron microscopy of retina from uninfected mice and mice infected with *Toxoplasma gondii* strain ME49. Semithin section of 2 μm from epoxy resin blocks, stained with toluidine blue, region of interest cut for ultramicrotomy (white bracket). (A–B) All layers of the retina of uninfected animals showed preserved structures and laminar organization, as well as RPE and choroid (Ch). (B) Ultrathin section of 70 nm of the retina of an uninfected animal showed preserved structures, such as the junction region of the outer segments with the RPE (white arrow), Bruch’s membrane (white bracket), and the choroid (Ch). (C–D) Infected mice showed alterations indicative of disorganization in the junctional regions between the photoreceptor segments and the RPE, and between the RPE and the choroid. (E–H) Infected mice were treated with a single dose of TST 20 μg/2 μL on the 10th day of infection and sacrificed on the 15th day. The eyes showed improvement in the regions of interest (white bracket) Scale bars: A, C, E, G, 50 μm; B and D, 2 μm; F and H, 1 μm.

### TST Plays a Pivotal Role in the Preservation of the Adhesion Areas of the Retina

To understand whether modulation of ZO-1 was involved in the protection of retinal tissue after TST treatment against *T. gondii* infection, as well as observed in vitro assays, infected animals were treated on day 10th, as described above, and were euthanized after 5 days of treatment. Retinal tissue was analyzed through immunolocalization of ZO-1 in cryo sections (Figure 6). The control group consisted of mice that were neither infected nor treated with TST. Control animals showed even labeling along the cell membranes, both in the outer limiting membrane (OLM) and in the BM. Both infected mice, on the 10th or 15th postinfection day, showed weak labeling with small dots. After 5 days of treatment, treated animals exhibited a visible recovery of ZO-1 labeling. Quantification of the fluorescence intensity of 20 random fields from six eyes showed that the treatment reestablished the basal levels of fluorescence of ZO-1 in animals without infection, thereby showing protection in the external blood-retinal barrier (Figure 6A, white arrows and 6B). This finding indicates that TST not only protects the tight junctions but also restores them after 5 days.

**Figure 6:**
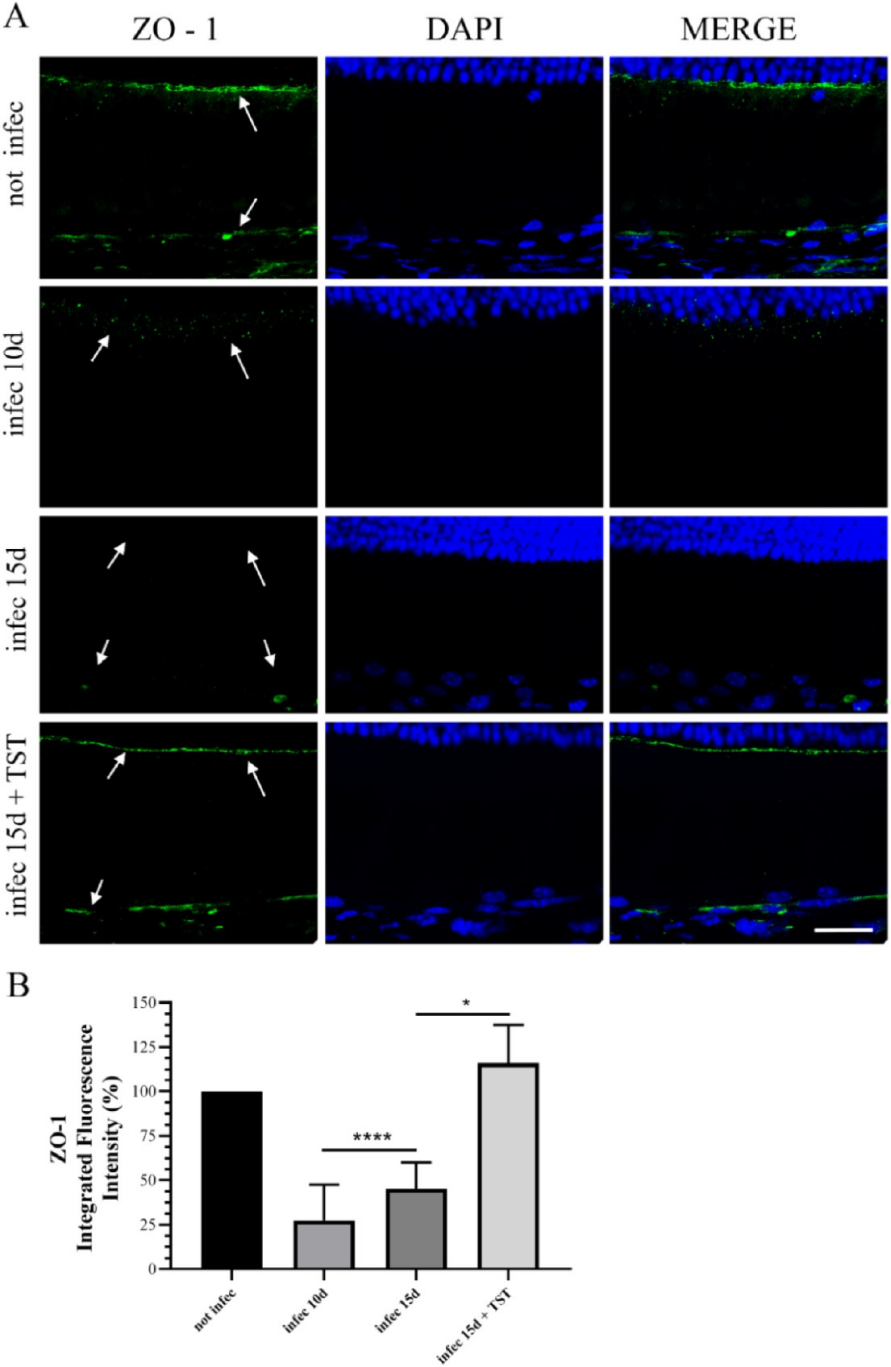
ZO-1 is a key protein in the pathophysiology of acute OT. In uninfected animals, intense staining for ZO-1 (green) was observed in the regions of the internal limiting membrane between the ONL layer and the inner segments, as well as between the RPE and the choroid. This staining indicates the integrity of the cell–cell junctions. In contrast, infected mice on the 10th and 15th days exhibited reduced ZO-1 staining, suggesting a significant disruption of the physical barrier formed by cell junctions due to parasite infection. The treatment of infected animals restored staining levels to those observed in the noninfected group. (B) A quantitative analysis of the integrated fluorescence intensity of the sections from animals stained for ZO-1 (green) was conducted. Statistical significance was assessed using Tukey’s multiple comparisons test, *P = 0.0428.

### Intravitreal TST-Regulated Micro- and Macroglia Activation Caused by *T. gondii* Infection

Microglia are resident immune cells of the retina that play a crucial role in pathogen control. However, an imbalance in its activity, synergized with other inflammatory factors can contribute to irreversible damage in this tissue.^12^ To determine whether TST regulates microglial activation, infected animals were treated on the 10th postinfection day and euthanized 5 days after treatment. Eyes were cryo-sectioned and processed for immunolocalization of the microglia Iba-1^+^ (Ionized calcium-binding adaptor molecule-1).

Immunofluorescence revealed that *T. gondii* infection induced intense labeling for Iba-1 in the retinal tissue of infected mice after 10 or 15 days of infection (Figure 7A). On the 15th postinfection day, it was possible to observe the migration of Iba-1^+^ cells among the layers, as well as their elongated cytoplasmic processes. Furthermore, in these infected mice, the number of Iba-1^+^ cells increased 7-fold compared to noninfected mice (Figure 7B). However, after TST treatment, downregulation of microglial activation led to a 3-fold decrease in the number of Iba-1+ cells compared with infected, untreated mice. These results indicated that a single injection of TST might reduce microglia reactivity. To determine whether it was related to negative feedback control of Iba-1 gene expression, mRNA levels and global protein quantification were evaluated. As illustrated in Figure 7A,C,D, the expression of Iba-1 decreased in response to the increased protein presence, as indicated by the intense labeling on the 10th or 15th postinfection days. After TST treatment, mRNA expression returned to near basal levels.

**Figure 7:**
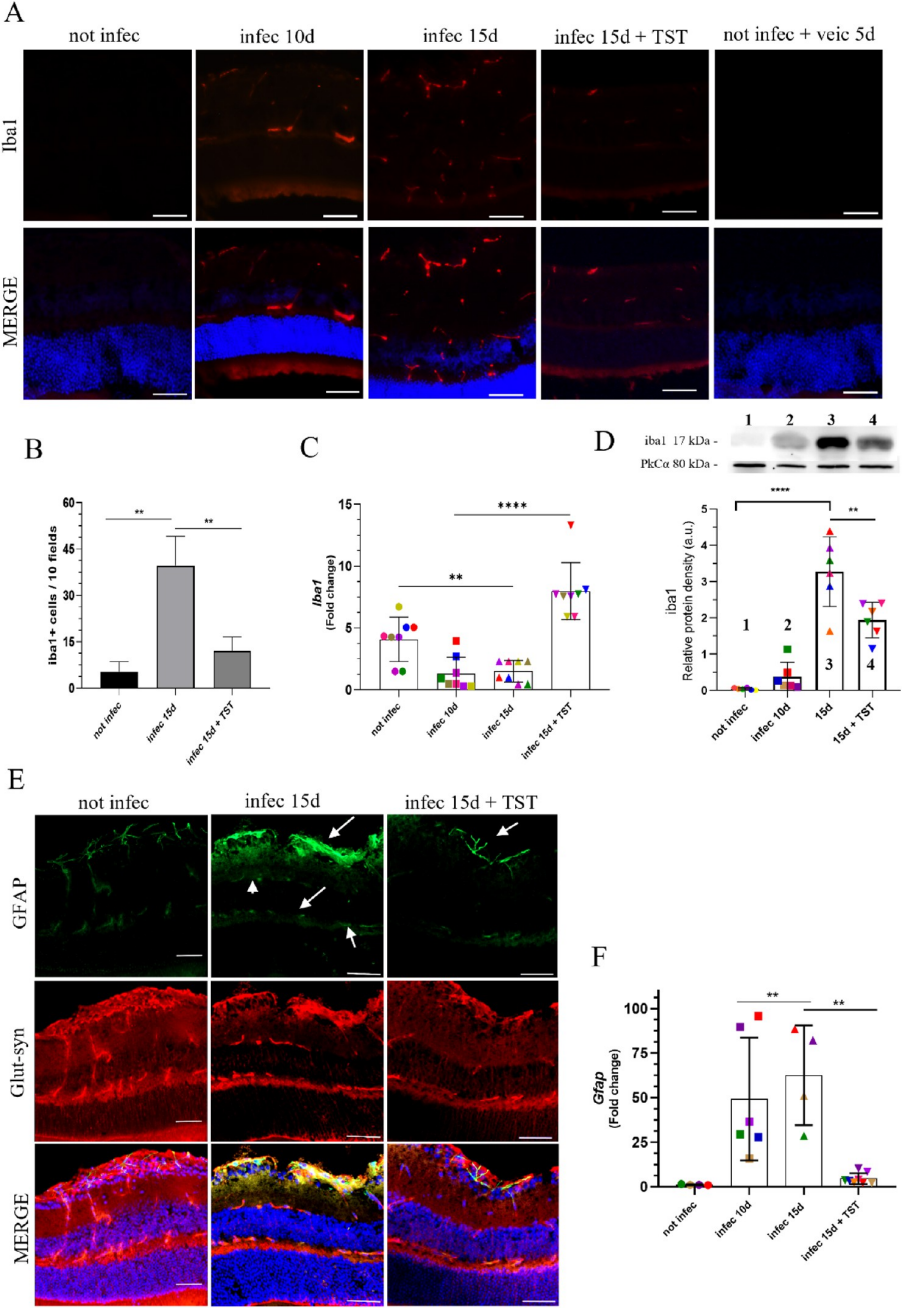
Indicative proteins of gliosis were altered, indicating high tissue reactivity in *T. gondii* infection, and TST treatment on the 10th day of infection restored them to basal levels. (A) Intravitreal treatment for 5 days restored Iba1 labeling levels like those in uninfected animals. (B) Quantification of Iba1-positive cells showed an increase in infected animals, whereas TST treatment reduced Iba1 labeling. (C) Infected groups showed lower *Iba1* gene expression than the uninfected control; TST treatment restored *Iba1* gene expression. Each point represents one eye. (D) As shown in A, Iba1 protein levels increased after 15 days of infection, and were reduced by 40% after 5 days of TST treatment. (E) Treatment for 5 days reduced GFAP protein labeling and restored Glut-syn distribution in Müller cells. (F) *Gfap* expression reflects the inflammatory response of astrocytes in the retina of animals infected with *T. gondii*. mRNA analysis showed that the gene expression increased after 10 and 15 days of infection and decreased with treatment. Each point represents one eye. Statistical significance was assessed using Tukey’s multiple comparisons test. (B) **P = 0.0053 (C) **P = 0.0098, ****P < 0.0001 (E) **P = 0.0040, ***P = 0.0003. Scale bar, 50 μm.

Macroglial cells in the retina, such as astrocytes and Müller cells, contribute to parasite control and the maintenance of tissue homeostasis. The GFAP protein is a marker of glial activation following nervous system damage, as well as in the retina.^36^ The glutamine synthetase protein (Glut-syn) decreases glutamate concentration, thereby protecting neurons from its toxic effects, and is mainly expressed in Müller cells.^36^ Because of that, macroglial GFAP and Glut-syn proteins were also evaluated in this ocular toxoplasmosis context.

Regarding GFAP, immunofluorescence analysis revealed that untreated infected animals exhibited increased labeling compared to noninfected animals (Figure 7E), which was also reflected in higher gene expression, as assessed by mRNA quantification (Figure 7F). In treated infected mice, TST modulated glial activation, preventing its increase compared with the nontreated infected group, whose gene expression levels and immunolabeling intensity were like those of uninfected animals. Analysis of the Glut-syn protein revealed that untreated infected mice exhibited morphological changes in the Müller cell processes across retinal layers. After TST treatment, recovery in the morphological distribution of Müller cell processes was confirmed by Glut-syn protein staining (Figure 7E). These findings suggest that TST regulates micro- and macroglial activation, thereby protecting retinal tissue against damage caused by *T. gondii* infection and by an imbalance in the immune response.

### TST Showed Anti-Inflammatory Effects in Ocular Toxoplasmosis

To understand whether key pro-inflammatory and regulatory cytokines in ocular toxoplasmosis (OT) would be altered after TST treatment, resulting in the preservation of retinal tissue, infected animals were treated on the 10th day postinfection with a single intravitreal injection, as described above. The animals were euthanized 5 days after treatment. Expression of *Tgfβ2*, *Il12*, *Il4*, and *Il17a* was evaluated through mRNA analysis. Each animal in each group was considered the sum of both eyes (Figure 8). Furthermore, cytokine protein levels were assessed by ELISA in macerated eyes from treated and untreated animals (Figure 9).

**Figure 8:**
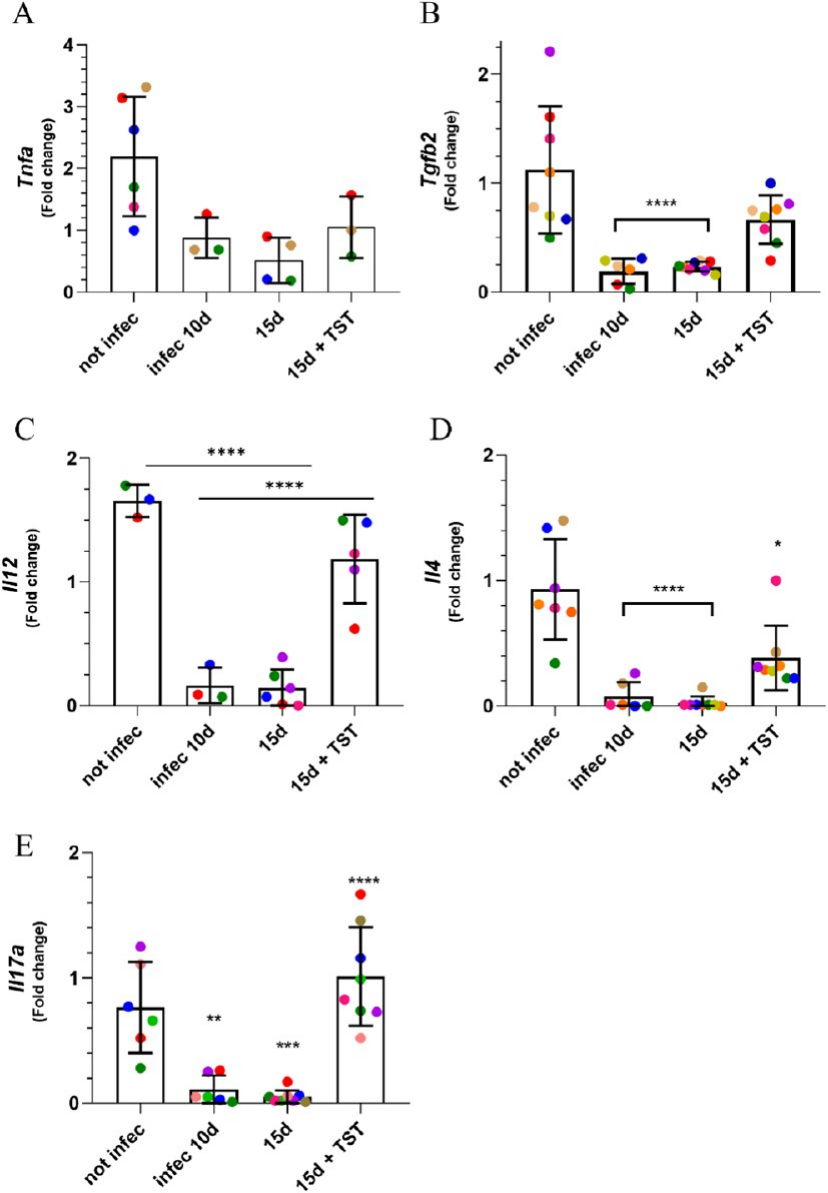
*T. gondii* modulates the expression of immunological factors in the eyes, and treatment with an HDAC6 inhibitor shows an immunomodulatory effect. (A) The pro-inflammatory factor *Tnfα* gene showed low expression on the15th day of infection and was not altered by the treatment. (B) The *Tgfβ2* showed the same pattern as the cytokines. Each animal in the groups was considered the sum of both eyes. (C-E) The cytokine genes *Il12*, *Il4,* and *Il17a* showed low expression in infected animals at the observed time points, and treatment recovered their levels to near baseline levels. Statistical significance was assessed using Tukey’s multiple comparisons test. (B) ****P < 0.0001 (C) ****P < 0.0001 (D) ****P < 0.0001 (E) **P = 0.0019, ***P = 0.0004, ****P < 0.0001.

**Figure 9:**
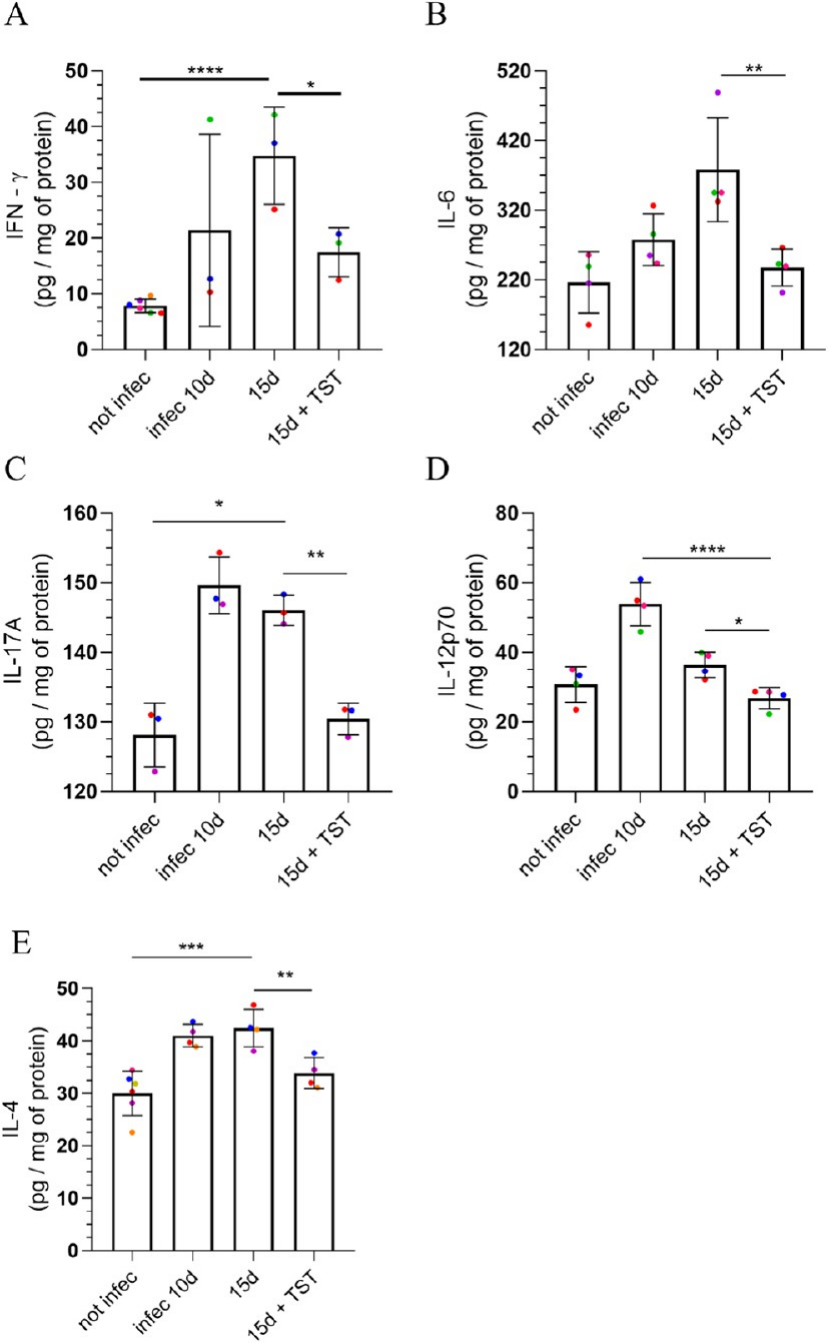
Cytokines secreted during the acute phase are modulated by HDAC6 inhibitor treatment, reducing the inflammatory response. All cytokines analyzed by ELISA assays showed the same pattern of increase at the time points analyzed, with a significant decrease with treatment. Each animal in the group was considered the sum of both eyes. Statistical significance was assessed using Tukey’s multiple comparisons test. (A) *P = 0.0233 ** *P* = 0.0082 (B) **P = 0.0090, ***P = 0.0018 (C) **P = 0.0052 (D) *P = 0.0475, ****P < 0.0001.

The expression of *Tgfβ2*, *Il12*, *Il4*, and *Il17a* showed a uniform transcriptional response on the 10th and 15th days postinfection, characterized by lower basal levels than in uninfected animals. The TST treatment led to partial recovery of gene expression levels (Figure 8B,C,E), except for *Tnf-α*, which declined without a regulatory effect after treatment (Figure 8A).

Analysis of cytokines at the protein level revealed that *T. gondii* infection led to a 45% increase in IFN-γ compared to baseline levels on the 15th day postinfection, with a 50% decrease with treatment (Figure 9A). Evaluation of pro-inflammatory cytokines showed increased IL-6 and IL-12p70 levels during the infection. There was a 28% and 75% increase in IL-6 on the 10th and 15th postinfection days, respectively, compared to the noninfected group. After 5 days of TST treatment, a 37% reduction was observed compared to the 15-day group (Figure 9B). 17% and 14% increase in the levels of the regulatory cytokine IL-17A on the 10th and 15th postinfection days, respectively, compared to uninfected animals. Notably, after 5 days of TST treatment, IL-17A secretion decreased, approaching levels observed in uninfected mice. In untreated infected mice, a significant 15% increase was observed on the 15th day of infection, compared to infected mice treated with TST (Figure 9C). An evaluation of IL-12p70 showed a 75% increase on the 10th day and a 18.42% reduction on the 15th day compared to the noninfected group. After TST treatment, a 26.34% reduction was observed compared with the infected, untreated group. In addition, there was a 13% decrease compared with basal levels in noninfected animals (Figure 9D). Concerning IL-4 levels, infected mice revealed a significant increase in this anti-inflammatory cytokine throughout the infection. On the 10th day, a 37% increase was observed, whereas on the 15th day, a 41% increase was found compared to the noninfected group. After 5 days of TST treatment, a 20% reduction was observed compared with the 15th day of infection (Figure 9E).

These results suggest that TST interferes with the expression and secretion of key cytokines involved in combating *T. gondii* infection, such as IFN-γ, IL-12, and IL-17, thereby contributing to a negative feedback loop that reduces the inflammatory response. Furthermore, this anti-inflammatory state may be associated with increased TGF-β2 expression. The regulation of pro-inflammatory cytokines IFN-γ, IL-12, and IL-17 should be another mechanism contributing to the preservation of retinal tissue against the toxic effects of exacerbated inflammation.

## Discussion

The use of intravitreal injections for antibiotics, antifungals, and antivirals in the treatment of ocular infectious diseases has become increasingly adopted due to their efficacy and safety profiles. This approach enables direct administration of antimicrobials into the vitreous humor, achieving high local drug concentrations and reducing systemic adverse effects.^37^ This therapeutic modality has been particularly impactful in cases of resistant infections or when systemic treatment is contraindicated.^38^ In cases of acute OT that do not respond to systemic antifolates, this approach has been a consistent clinical strategy.^39,40^ The rise in toxoplasmosis outbreaks over the past 20 years, driven by strains with high genetic diversity that often cause eye problems, underscores the urgency of developing new treatments. The drug repositioning strategy for OT might reduce the time and cost of finding new therapies. Furthermore, the investigation of novel alternatives for treating OT via the intravitreal route has revealed the efficacy of HDAC6 inhibitors. In this regard, TST has been evaluated in vitro as a potential anti-*T. gondii* candidate, along with other HDAC inhibitors.^33,41^

In the in vitro assays, TST did not exhibit cytotoxicity in RPE cells, even at high concentrations, such as 262 μM, which is 5 × 10^2^ times higher than the IC_50_ for *T. gondii* tachyzoites.^33^ In addition, the findings regarding safety were consistent with those from previous studies using other mammalian cell types, such as fibroblasts and renal epithelial cells.^34^ The toxicity evaluation in a healthy *in vivo* model showed that the intravitreal injection of 20 μg/2 μL of TST did not result in any changes by the procedure 5 days after injection, indicating the safety of TST for retinal tissue in subsequent intravitreal chemotherapy tests. In a study by Choi et al. (2019),^41^ using an optic nerve crush model in Sprague–Dawley rats, an intravitreal injection of 26.8 μg/4 μL TST stabilized primary cilia and reduced retinal ganglion cell apoptosis. In a zebrafish model, TST also restored visual function and retinal morphology.^42^

By elucidating the protective mechanisms conferred by TST on infected cells with *T. gondii*, we identified that TST prevents actin recruitment for parasitophorous vacuole formation and consequently hinders parasite invasion. The enzyme HDAC6 deacetylates multiple sites on various proteins, including cortactin, a cytoskeletal component that interacts with ZO-1 via its SH3 domain. HDAC6 inhibition contributes to increase stability of the cortical actin cytoskeleton.^43–45^ Therefore, the increased stability of the actin cytoskeleton induced by TST treatment may directly account for the observed reduction in tachyzoite invasion. In addition, other protective effects of TST could be observed in remaining cell–cell junction proteins, as evidenced by anti-ZO-1 and antipan-cadherin antibodies in ARPE-19, suggesting an autocrine signaling mechanism driven by the cell type’s intrinsic characteristics. This TST effect on ZO-1 was confirmed by in vivo assays. The ultrastructural data showed that infected animals had alterations in Bruch’s membrane, and that the adhesion junctions between RPE microvilli and the basal lamina were dysmorphic. In treated infected mice, the preservation of this barrier was demonstrated. In immunofluorescence assays on the 15th postinfection day, ZO-1 staining was not detectable in either the outer or inner limiting membrane. However, in treated infected mice, recovery of ZO-1 labeling was observed. These findings indicate that TST treatment not only protects but also restores zona occludens protein during the acute period of OT. This might explain the protective event observed in the subretinal region in all treated animals.

Previous studies have described the expression of angiogenic factors in OT, including increased *Vegf* expression and decreased *Thbs1*.^23^ The HDAC6-Hsp90 axis regulates the *VEGF* expression.^45^ Once Hsp90 deacetylation is inhibited, its activation and complexation with target proteins, such as HIF-1α, are impaired.^46,47^ It has been demonstrated that *T. gondii* can modulate host factors, including the stabilization of the HIF subunit, which leads to the activation of HIF-1α even under normoxic conditions.^48^ In the present study, these findings about increased *Vegf* expression were reproduced in infected ARPE-19 cells after 24 h of infection. However, TST treatment reverses the increase in *VEGF* levels without affecting *THBS1* expression. Regarding *HIF-1α*, *T. gondii* infection did not alter its expression. However, *HIF-1α* expression was significantly downregulated by TST treatment in infected ARPE-19 cells, suggesting that this effect may be related to HDAC6 inhibition. Therefore, it is probable that the protective effects observed in the RPE might not be due to the modulation of the antiangiogenic pathway of TSP1, but rather to the suppression of VEGF, through the inhibition of the HDAC6-Hsp90 axis.^45^ This axis has been investigated as a potential therapeutic alternative for wet age-related macular degeneration (AMD).^45^

Another factor in the HDAC6-Hsp90 axis that may help minimize damage to retinal tissue in infected animals is the inhibition of HSF1. HSF1 is considered the master regulator of molecular chaperone synthesis in response to the accumulation of misfolded proteins.^49^ The increased concentration of the chaperones in the cytosol regulates the HSF1 activation cycle itself.^50^ Although the transcription factor HSF1 has conventionally been associated with the response to heat stress, there is evidence that other stress factors also activate HSF1, as previously demonstrated.^51^ For the first time in the literature, the data presented here showed that *T. gondii* infection significantly increased *HSF1* expression by 52% compared with uninfected ARPE-19 cells, and TST treatment downregulated *HSF1* expression in infected ARPE-19 cells by 77% compared with nontreated cells. In this study, *HSF1* expression increased to high levels in infected ARPE-19 cells, which might directly contribute to the maintenance and persistence of the parasite within the cell. Therefore, *HSF1* downregulation may exert a protective effect on retinal tissue.

In this study, histopathological and ultrastructural analyses of retinas from *T. gondii*-infected animals revealed RPE damage, subretinal migration, cell displacement between the INL and CGL layers, and marked choroiditis. None of these changes were observed after treatment with a single TST injection, either after 3 or 5 days postinfection, indicating a high level of protection of the morphology compared to the abnormalities seen in untreated infected animals. Surprisingly, this protection was also not associated with a decrease in retinal parasite load in infected mice. However, a reduction in BAG1, a bradyzoite-specific marker, was observed after TST treatment. Nevertheless, previous studies in vitro showed the anti-*T. gondii* activity of TST.^33^ Perhaps, additional TST doses or combination with standard therapy may be required to achieve a significant reduction in parasite load.

The anti-inflammatory effects, which are essential for tissue preservation during an acute infection of the sensory retina, were also investigated. Iba-1 is involved in increased interferon-γ expression and the antimicrobial response to intracellular pathogens such as *T. gondii*, with an M1 profile.^52^ On the 15th postinfection day, nontreated infected mice showed many positive cells, indicating microglial reactivity. After TST treatment, a few labeled cells appeared, consistent with transcriptional negative feedback on *Iba1* caused by increased mRNA expression. The lower number of Iba-1^+^ cells observed in this study indicates that the treatment decreased microglial activation and reduced the M1 profile.

In addition, TST reduced both immunofluorescence labeling and *Gfap* expression, showing an effect that modulates astrocyte activation. The treatment restored the distribution of Glut-syn in Müller cell segments, thereby favorably modulating the cells that compose the inner blood-retinal barrier. It is noteworthy that Müller cells metabolize the excess glutamate secreted by neurons, converting it to glutamine and exporting it back to the neurons.^36^ The imbalance of this process leads to retinal pathologies.^53^ There are no reports in the literature of glial activation after intravitreal TST treatment, except for studies using systemic TST treatment in models of Parkinson’s disease, which have shown decreased expression of GFAP and Iba-1.^54–56^ These data reinforce that TST can modulate different types of macroglia in the retina against pathogen infection. The effect of intravitreal TST on these two cellular proteins in the acute OT was here demonstrated for the first time.

The treatment with TST also modulated the expression of immunoregulatory factors related to the response to *T. gondii* infection, such as *Tgfβ2*, which plays a crucial role in shaping the immune privilege of the retina and is constitutively expressed and secreted by RPE cells.^57^ Treatment with TST restored the expression of *Tgfβ2* in treated infected animals to the levels observed in uninfected animals, in addition to restoring the expression of *Il4*, *Il12*, and *Il17*, which were reduced in infected animals compared to baseline levels. On the other hand, the treatment did not have the same effect on *Tnfα*, which remained at low levels in all conditions observed. Previous work has shown that the increase in *Il17* levels observed with TST treatment may be related to modulation of *Tgfβ2*, which enhances Th17 cell differentiation.^58^ In an in vitro study with Müller cells infected with *T. gondii*, *Tgfβ2* expression was downregulated. In this study, TST treatment increased *Tgfβ2* expression and provided tissue protection in all animals.

OT inflammatory microenvironment is characterized by the coordinated activation of the Th1 and Th17 responses, two key pathways of adaptive immunity strongly associated with retinal immunopathology.^59^ The production of IL-12, a cytokine essential for Th1 polarization and IFN-γ induction, showed a pattern compatible with the acute phase of infection, reflecting an initial robust response that subsequently became more controlled, which might be related to the exhaustion or downregulation of inflammatory pathways. This dynamic is consistent with previous studies that describe the protective role of the Th1 response in controlling parasite replication in the eye, as well as its association with retinal tissue destruction when not properly regulated.^60,61^

Activation of the Th17 pathway was evidenced by the sustained increase in IL-17A, a cytokine strongly implicated in disrupting the blood-retinal barrier and recruiting neutrophils and monocytes.^62^ The persistent rise in IL-6 throughout the infection supports the hypothesis that the Th17 response contributes significantly to acute ocular inflammation in toxoplasmosis, as also described in murine ocular lesions with diabetic retinopathy, which exhibit infiltration of Th17 cells.^63^ Intravitreal TST treatment significantly modulated this inflammatory environment. A reduction in cytokines associated with the Th1 and Th17 pathways was observed, suggesting possible interference by TST with retinal resident cells, such as microglia and Müller cells. It is known that HDAC inhibitors, such as TST, can act on NF-κB and STAT3-dependent transcription pathways, reducing the expression of pro-inflammatory genes.^64,65^ The decrease in IL-6 and IL-17A after treatment supports the hypothesis that TST limits the perpetuation of the Th17 response, creating a less harmful environment for neural tissue. TST can attenuate inflammation without abolishing local immunity, which is critical in ocular infectious diseases, where the balance between immunity and immunopathology is essential. Reduced IL-12 levels and restored IL-4 suggest a shift in the ocular immune response, reducing Th1 activation and possibly inducing a regulatory phenotype. These results suggest TST immunomodulation is key to maintaining ocular immune privilege and protecting retinal tissue from excessive inflammation caused by *T. gondii* infection. In clinical practice, intravitreal TST may prevent neovascular lesions in OT and, combined with oral antifolates, could offer a new treatment for protecting retina sensory tissue.

## Conclusion

Screening for novel compounds remains essential in acute OT, given the limited efficacy of current therapies and the risk of refractory disease. Our findings demonstrate that selective HDAC6 inhibition confers multifaceted protection, with a central role in preserving the integrity of the outer blood–retinal barrier. These results underscore the need for further studies to elucidate the molecular mechanisms underlying HDAC6i-mediated tissue protection and immune modulation. Moreover, the administration route employed here may represent a promising therapeutic strategy for cases refractory to standard oral treatment.

## Supplementary Material



## Notes

Experimental details and materials and methods can be found in the Supporting Information (PDF)
