# Supplementary material for: Intravitreal Administration of a Selective HDAC6 Inhibitor Prevents Retinal Damage Progression in the Acute Ocular Toxoplasmosis Model
Source: ACS Infect Dis. 2026 Jan 14;12(2):665–78. doi: 10.1021/acsinfecdis.5c00818 (PMC12910585; doi:10.1021/acsinfecdis.5c00818)
Supplement: Supplementary file 1 [file id5c00818_si_001.pdf]

Supporting Information

Intravitreal administration of a selective HDAC6 inhibitor prevents retinal damage progression in the acute ocular toxoplasmosis model

Carlla Assis Araujo-Silva[a,b], Milena Ribeiro Peclat-Araujo[a,b], Vanderlei da Silva Fraga-Junior[c], Thuany Prado-Rangel[a,b], Dio Pablo Alexandrino-Mattos[d], Claudia Farias Benjamim[c], Christina Maeda Takiya[d], Wanderley de Souza[a,b], and Rossiane Claudia Vommaro*[a,b]

[a] Laboratório de Ultraestrutura Celular Hertha Meyer, Centro de Pesquisa em Medicina de Precisão, Instituto de Biofísica Carlos Chagas Filho, Universidade Federal do Rio de Janeiro, Cidade Universitária, Rio de Janeiro, RJ, 21941-599, Brasil

[b] Instituto Nacional de Ciência e Tecnologia em Biologia Estrutural e Bioimagens, Universidade Federal do Rio de Janeiro, Brasil,

[c] Laboratório de Imunologia Molecular e Celular, Instituto de Biofísica Carlos Chagas Filho, Universidade Federal do Rio de Janeiro, Cidade Universitária, Rio de Janeiro, RJ, 21941-904, Brasil

[d] Laboratório de Imunopatologia, Instituto de Biofísica Carlos Chagas Filho, Universidade Federal do Rio de Janeiro, Cidade Universitária, Rio de Janeiro, RJ, Brasil

[e] Laboratório de Terapia Gênica e Vetores Virais, Centro de Pesquisa em Medicina de Precisão, Instituto de Biofísica Carlos Chagas Filho, Universidade Federal do Rio de Janeiro, Cidade Universitária, Rio de Janeiro, RJ, 21941-599, Brasil

Corresponding author: Rossiane Claudia Vommaro – vommaro@biof.ufrj.br

Keywords: Retina pathology, Ocular disease, Immunomodulation, Experimental chemotherapy, Tubastatin A

Supporting Information Figure 1

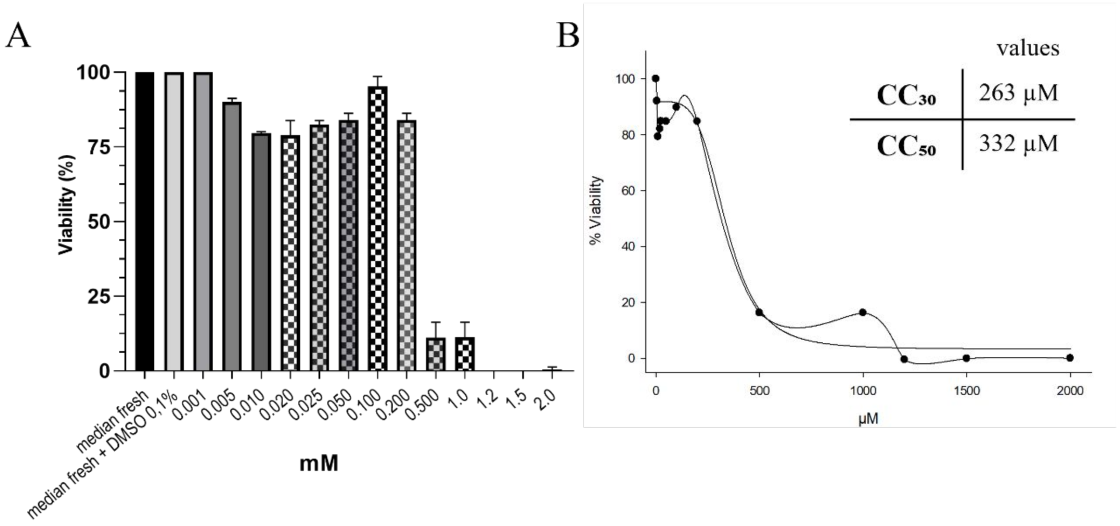

Supporting Information Figure 1: MTS assay in ARPE-19 cells incubated for 72 hours with TST at different concentrations. (A) Cell viability data were plotted using GraphPad Prism 8. The viability of RPE cells was not compromised until 0,2 mM. (B) The nonlinear regression curve used to determine CC_{30} and CC_{50} values was generated in SigmaPlot 12 from experimental triplicate data.

Supporting Information Figure 2

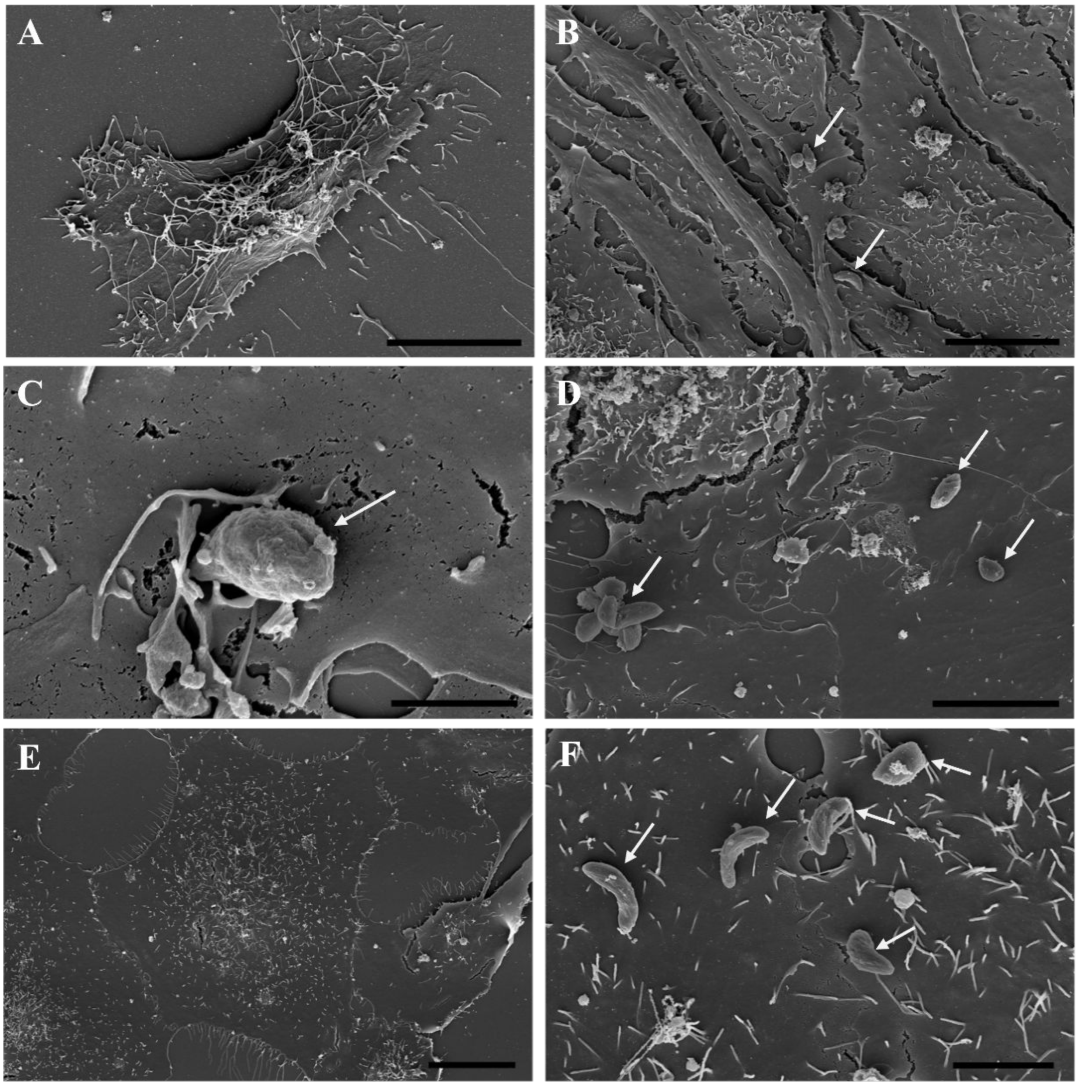

Supporting Information Figure 2: Invasion assay in ARPE-19 cells pretreated for 48 hours with TST. An invasion assay was prepared for SEM: (A) Cells in fresh medium. (B-C) Cells without pretreatment were infected with tachyzoites for 1 hour. White arrows indicate parasites actively invading cells. (D) Cells were pretreated and infected for 1 hour. White arrows indicate parasites dispersed in diverse positions. (E) Cells after 2 hours of interaction with tachyzoites, without pretreatment. The majority of tachyzoites have already completed cell invasion. (F) Cells pretreated and infected for 2 hours showed tachyzoites dispersed on the cell surface. Scale bars: A- 15 µm; B- 15 µm; C- 2 µm, D- 10 µm; E- 25 µm; F- 5 µm.

Supporting Information Figure 3

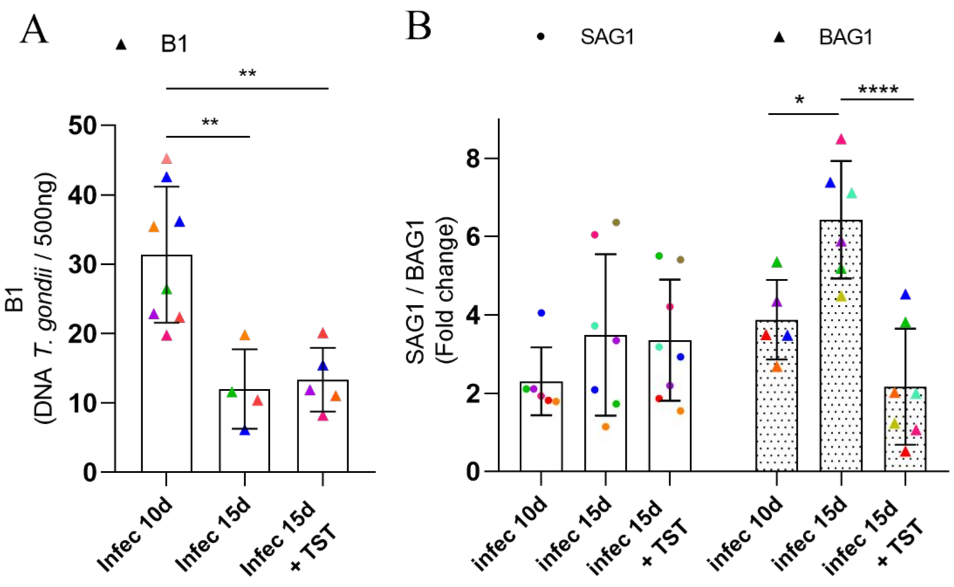

Supporting Information Figure 3: Quantification of *Toxoplasma gondii* load and stage-specific markers in ocular tissue following infection and treatment. (A) Genomic DNA quantification of *T. gondii* B1 gene by qPCR from individual mouse eyes at 10 days post-infection (infec 10d), 15 days post-infection (infec 15d), and 15 days post-infection followed by TST treatment (infec 15d + TST). Each data point represents one eye. (B) Relative expression levels of the tachyzoite marker SAG1 and bradyzoite marker BAG1, quantified by RT-qPCR from total RNA extracted from individual eyes. Data are presented as fold change relative to the control condition (indicated by reference), and each point represents one eye. Statistical significance was assessed using Tukey's multiple comparisons test. (A) **P=0.0079, *P=0.0348 (B) *P= 0.0220, ****P <0,0001.

Materials and Methods

Dilution of the TST compound

Tubastatin A (TST) (Sigma-Aldrich) was solubilized in dimethylsulfoxide (DMSO, Merck). The final concentration of DMSO never exceeded 0.1% (v/v) *in vitro* and *in vivo* assays.

Host cells, parasites, and mice

Normal Human Neonatal Dermal Fibroblasts (NHDF-Neo) (Lonza®) and ARPE-19 (BCRJ), a human retinal pigment epithelium (RPE) cell line, were cultivated according to Freshney, R. Ian, 2005 [1]. ME49-GFP-Luciferase strain of *T. gondii* (referred to in the whole text as ME49-GFP (kindly given by Dr. John Broothroyd) was cultivated in NHDF. For *in vitro* assays of tachyzoite interaction with ARPE-19, a 5:1 MOI and 5 μM TST were used for 24 hours, depending on the assay. To assess whether the HDAC6 inhibitor interfered with the active entry of *T. gondii* into cells, ARPE-19 cells were pre-treated with 5 μM TST for 48 hours and then challenged with tachyzoite at a MOI of 5:1 for 1 or 2 hours in fresh medium. For all *in vivo* assays, 8- to 16-week-old C57BL/6 male mice were infected intraperitoneally with 10^4 tachyzoites.

The author`s statement of the ethics approval process for the use of animals.

The authors declare that all the experimental protocols using mice were submitted to and approved by the Ethics Committee for the Use of Animals (CEUA) in Scientific Experimentation of the Health Sciences Center of the Federal University of Rio de Janeiro, registered at the National Council for the Control of Animal Experimentation (CONCEA) under the process number 01200.001568/2013-87, and the license No. A13/25-A35-24-067-20.

Cytotoxicity assay of TST in ARPE-19

To assess cell viability, we used the MTS method (3-(4,5-dimethylthiazol-2-yl)-5-(3-carboxymethoxyphenyl)-2-(4-sulfophenyl)-2H-tetrazolium, inner salt) (Promega) on ARPE-19 monolayers in three independent experiments to evaluate the cytotoxicity of TST. In total, 10^4 cells were seeded in 96-well plates for 48 hours before the experiment and treated for 72 hours. TST was tested at different concentrations: 0.001, 0.005, 0.010,

0.020, 0.025, 0.050, 0.100, 0.200, 0.500, 1.0, 1.2, 1.5, and 2.0 millimolar. Cytotoxicity was calculated considering the percentage of viable cells. The CC_{30} and CC_{50} values were determined by fitting a nonlinear regression curve using SigmaPlot 12 software.

Intravitreal injection

Animals were anesthetized by intraperitoneal injection of a non-lethal mixture of ketamine (75 mg/kg) and xylazine (5 mg/kg). Pupils were dilated with 10 µL of 1% tropicamide (Alcon) eye drops, applied for 5 minutes, followed by topical administration of 0.5% proxymetacaine hydrochloride (Alcon). Before the intravitreal injection, a small incision was made in the sclera at the pars plana region (located between the cornea and the retina, in the temporal/superior quadrant of the eye) using a 30 G needle. Intravitreal injections were performed using a 5 µL Hamilton syringe with a straight 33 G needle, delivering 2 µL of TST at a concentration of 10 µg/µL diluted in PBS. To ensure retinal integrity and avoid structural damage, fundus examination was performed after the procedure. The injection needle was held in place for 30 seconds post-injection to minimize reflux before removal. All procedures were conducted under a surgical microscope (OPMI 1, Carl Zeiss®) or stereomicroscope (NIKON). Following the injection, animals were closely monitored until full recovery from anesthesia and subsequently returned to the animal facility. The treatment timeline used is described in Figure S4 A and B.

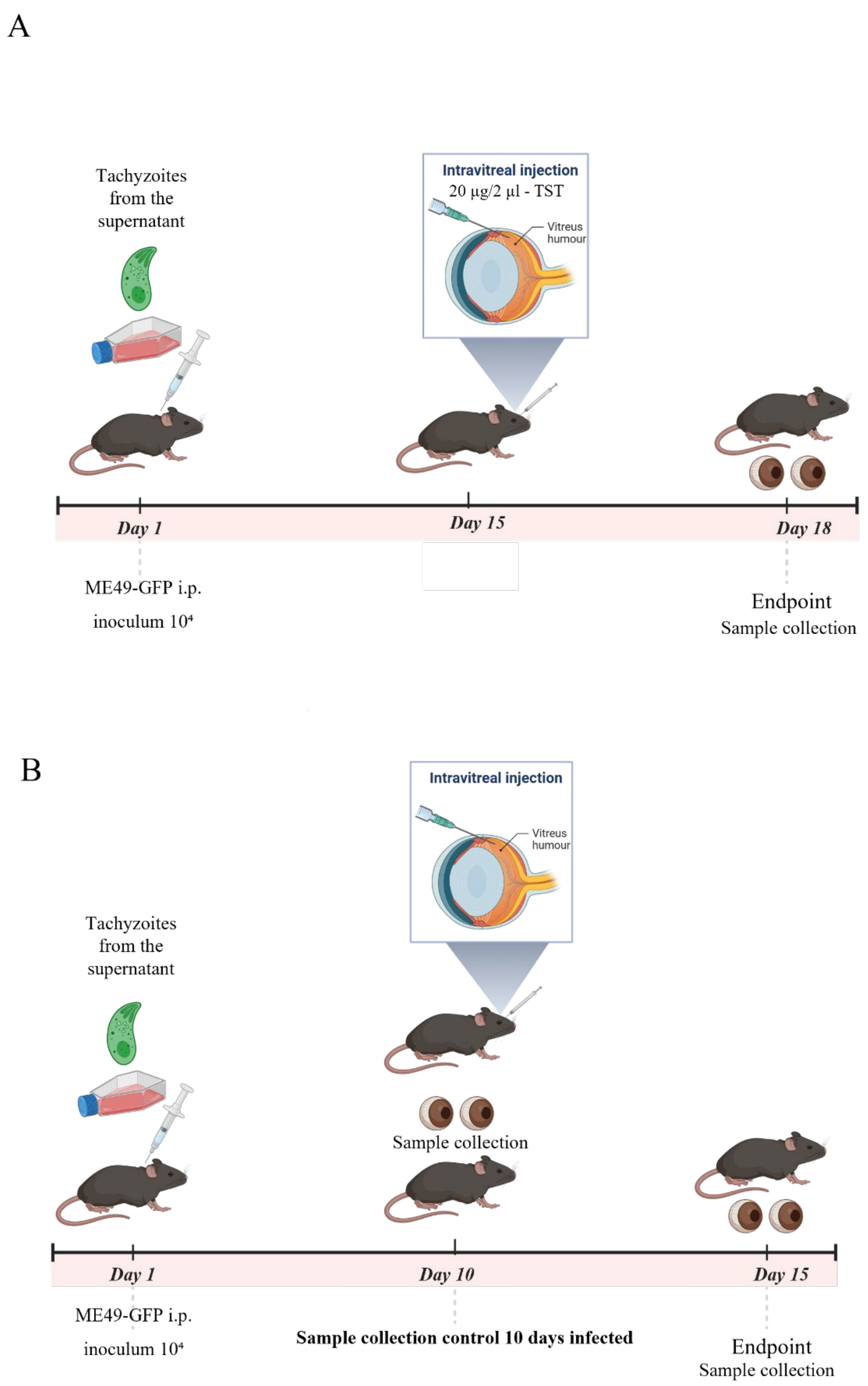

Supporting Information Figure 4: Timeline of intravitreal treatment administration. (A) The inoculum of 10^4 tachyzoites was administered intraperitoneally in 100 µl of PBS. After 15 days of infection, TST was administered. After 18 days of infection, the treated animals were euthanized, and their eyes were processed according to the analysis. (B) The inoculum of 10^4 tachyzoites was administered intraperitoneally in 100 µl of PBS. 10 days after infection, TST was administered. After 15 days of infection, the treated animals were euthanized, and their eyes were processed according to the analysis. Eyes of the control animals were also collected on the 10th and 15th days after infection.

Intracardiac Perfusion

For vascular bed flushing and tissue fixation, adult male mice (approximately 25 g) were anesthetized via intraperitoneal injection of an overdose of ketamine and xylazine, administered in accordance with the AVMA Guidelines for the Euthanasia of Animals (2020) and the institutionally approved animal use protocol (CEUA, 067/20). Following thoracic cavity opening, a 26-G catheter was inserted into the left ventricle, and the pulmonary artery was transected. The vascular system was flushed with 0.9% saline solution using a peristaltic pump (MILAN bp600®) at 80 mmHg. Subsequently, perfusion was performed with either 2.5% glutaraldehyde (EMS) in 0.1 M phosphate buffer (for ultrastructural analysis) or 4% paraformaldehyde (Sigma Aldrich) in 0.1 M phosphate buffer (for immunohistochemical analysis) for 5 minutes.

Histological Processing of Ocular Tissue

Following intracardiac perfusion with 2.5% glutaraldehyde, the eyes were enucleated and post-fixed overnight in the same fixative. Samples were dehydrated in graded ethanol (Merck) (50–100%) and incubated for 2 hours in a 1:1 ethanol:Technovit® 7100 (Kulzer Technik), followed by immersion in pure historesin overnight at 4°C. Tissues were embedded in Technovit® 7100 with the catalyst and polymerized at room temperature for 24 hours. Semi-thin sections (2 µm) were cut with a Reichert ultramicrotome (Leica), mounted on 1% gelatin-coated slides, stained with 1% toluidine blue, and sealed with Entellan® (Merck) for light microscopy. For paraffin embedding, tissues were dehydrated in ethanol (50–100%), cleared in xylene (Merck), and infiltrated with Paraplast (Leica) at 60°C. Sections (5 µm) were obtained using a rotary microtome (Leica), stained with hematoxylin and eosin (H&E), and mounted with Entellan® (Merck).

Electron Microscopy

For transmission electron microscopy (TEM), eyes were enucleated and post-fixed with 1% osmium tetroxide for 45 minutes. Dehydration was performed in acetone followed by infiltration in epoxy resin (12 h each step). Samples were polymerized at 60°C for 3 days. 70nm sections were contrasted with uranyl acetate and lead citrate. Samples were observed in an HT 7800 (Hitachi) TEM. For scanning electron microscopy (SEM), cells were fixed with 2,5% glutaraldehyde (EMS) and post-fixed with 1% osmium tetroxide (EMS). Samples were dehydrated in ethanol and critical point dried in a Balzers apparatus. Samples were coated with 5 nm of platinum and examined using the Quattro S SEM (Thermo Fisher Scientific) at CENABIO III facility (UFRJ).

Immunofluorescence

Animals were subjected to intracardiac perfusion 5 days after TST injection. Eyes were fixed by overnight immersion in 4% paraformaldehyde. Eye orientation was ensured by a small needle incision in the superior cornea. For cryo-sectioning, the cornea, iris, and lens were removed after fixation, and the eyecups were immersed overnight in 30% sucrose for cryoprotection. Samples were then embedded in optimal cutting temperature (OCT) compound (Tissue-Plus™ OCT, Fisher Healthcare™), frozen, sectioned at 14 µm, and collected onto slides coated with 6% silane (Sigma Aldrich). Slides were stored at –20°C for subsequent immunofluorescence labeling with different cellular markers. ARPE-19 cells on coverslips were fixed and processed following the same immunolocalization protocol used for the tissue samples. Antigen retrieval was performed by immersing the slides in 10 mM citrate buffer at 60°C for 5 minutes, followed by permeabilization in 1% Triton X-100 (Sigma Aldrich) in TBS for 15 minutes. After permeabilization, all samples were incubated in 100 mM ammonium chloride blocking solution and in TBS-T blocking buffer (20 mM Tris, 150 mM NaCl, 0.05% Tween-20, pH 7.3) containing 5% bovine serum albumin (BSA) (Sigma Aldrich). Samples were then incubated overnight at 4 °C in a humidified chamber with primary antibodies (Supporting Information Table 1). The next day, the sections were incubated for 2 hours with Alexa Fluor-conjugated secondary antibodies (Alexa 546, 568, or 647; Invitrogen™) diluted 1:800. Negative controls were performed by omitting the primary antibody. Nuclear staining was performed with DAPI (0.5 µg/mL; Thermo Fisher®) for

30 minutes. Slides were mounted with ProLong™ Gold Antifade Mountant (Invitrogen®). Images were acquired with ELYRA PS1 or LSM 900 on an Axio Observer microscope and processed with ZEN Blue 3.9 (Zeiss).

Supporting Information Table 1

**Antibody**	**Enterprise**	**Dilution**
α - Iba1 (mouse)	Invitrogen	1:1000
α - GFAP (rabbit)	Sigma-Aldrich	1:1000
α - Glutamin-Synthetase (mouse)	Santa Cruz Biotechnology	1:1000
α - ZO-1 (rabbit)	Invitrogen	1:500
**Probe**	**Enterprise**	**Dilution**
ActinRed™ 555	Thermo Scientific	1:50
DAPI (5mg/ml)	Thermo Fisher	1:800

Fluorescence intensity quantification and image processing

Fluorescence images were acquired using a widefield inverted microscope (Zeiss). The fluorescence intensity was quantified by analyzing 10 random fields from 4 experimental animals. For the *in vitro* analysis, 10 random fields were captured using a 40× objective in 3 independent experiments. The measurements were performed using Zen Blue 2.3 software (Zeiss).

Western blotting

Infected ARPE-19 cells were treated and infected with tachyzoites at different time points with 5 µM TST, then scraped and resuspended in lysis buffer containing 0.1% protease inhibitor cocktail (Sigma Aldrich). Membranes were incubated with α-ZO1 (1:1000, Sigma), α-Pan-cadherin (1:1000, Abcam) and α-Iba1 (1:500, Invitrogen) overnight at 4°C, depending on the assay. PKCα was used as the loading control (1:500, Santa Cruz) for 1 hour. Membranes were incubated with peroxidase-conjugated α-mouse or α-rabbit IgG 1:4000 (Promega). Membranes were exposed to ECL reagent (Promega) and visualized using ImageQuant LAS 500 (GE Healthcare Life). Band intensities from

three independent experiments were quantified using NIH ImageJ, with protein amounts normalized to the loading control.

Cytokine assay - ELISA

Eyes were collected, macerated in lysis buffer containing a 0.1% protease inhibitor cocktail (Sigma Aldrich), and cryopreserved at –80 °C. The total protein was quantified with a Pierce BCA Protein Assay Kit (Thermo Scientific) and used to normalize the results. Eye homogenates were used for the cytokine quantification (IFNγ/DY285B-05, IL-4/DY404, IL-6/DY406, IL-12p70/DY419, IL-17/ DY421) using R&D Systems ELISA Kits. The results were expressed as cytokine/mg of protein.

RNA Isolation, cDNA Synthesis, qRT-PCR and gDNA Extraction

Analyses were performed from macerated whole eyes or cultured cells, RNA was extracted using TRIzol® (Thermo Fisher), quantified, and assessed for purity with a NanoDrop BioChrom (Harvard Bioscience). cDNA was synthesized from 1 μg RNA using GoScript™ Reverse Transcriptase (Promega). DNA was isolated with the DNeasy Blood & Tissue Kit (Qiagen), eluted in 50 μL TE buffer, and stored at 4 °C. qPCR was performed using SyGreen Mix/ROX (PCRBIOSYSTEMS) on a QuantStudio™ 3 (Applied Biosystems). The murine and human β-actin genes were used as endogenous controls. The qPCR primers (Exxtend) that were used are detailed in Supporting Information Table 2. Relative gene expression was calculated using the comparative cycle threshold (Ct) method (ΔΔCt) or a standard curve.

Supporting Information Table 2: Primers

T. gondii:	
B1	*F* GGAACTGCATCCGTTCATGAG<br>*R* TCTTTAAAGCGTTCGTGGTC
SAG-1	*F* CCCACACTGATGTCGTTCTT<br>*R* CTTGAGAGTGAAGTGGTTCTCC
BAG-1	*F* CCTCGAAAGAAGCGGAGAAA<br>*R* GATTCCGTCGGGCTTGTAAT
**Murine:**	
Iba1	*F* GTCCTTGAAGCGAATGCTGG<br>*R* CATTCTCAAGATGGCAGATC
Gfap	*F* TCCTGGAACAGCAAAACAAG<br>*R* CAGCCTCAGGTTGGTTTCAT
Gapdh	*F* AGGTCGGTGTGAACGGATTTG<br>*R* TGTAGACCATGTAGTTGAGGTCA
β- Actin	*F* GAGGTATCCTGACCCTGAAGTA<br>*R* CACACGCAGCTCATTGTAGA
Tnfα	*F* TCTCATCAGTTCTATGGCCC<br>*R* GGGAGTAGACAAGGTACAAC
Tgfβ2	*F* CTTCGACGTGACAGACGCT<br>*R* GCAGGGGCAGTGTAAACTTATT
Il12	*F* GAGCACTCCCCATTCCTACT<br>*R* GCATTGGACTTCGGTAGATG
Il17	*F* ACTCTCCACCGCAATGAAG<br>*R* TTCAGGACCAGGATCTCTTG
Il4	*F* GGTCTCAACCCCCAGCTAGT<br>*R* GCCGATGATCTCTCTCAAGTGAT
Vegfa	*F* AGGCTGCTGTAACGATGAAG<br>*R* TCTCCTATGTGCTGGCTTTG
Hif1α	*F* ACCTGGCAATGTCTCCTTTAC<br>*R* CCAGTGACTCTGGACTTGATTC
**Human:**	
VEGFA	*F* CTGTCTTGGGTGCATTGGAG<br>*R* ACCAGGGTCTCGATTGGATG
HIF1A	*F* GTACCCTAACTAGCCGAGGAAGAA<br>*R* GTGAATGTGGCCTGTGCAGT
THBS1	*F* AAAGGATAATTGCCCCAACC<br>*R* CGGTCTCCCACATCATCTCT
HSF1	*F* TGAAAAGTGCCTCAGCGTAGCC<br>*R* TGCTCAGCATGGTCTGCAGGTT

Statistical Analysis

Statistical analyses were performed in GraphPad Prism 8 (GraphPad Software). A two-tailed *t*-test was used for pairwise comparisons. Multiple comparisons were performed by one-way ANOVA followed by the post hoc Tukey's test, with $P < 0.05$ considered statistically significant. The results were expressed as means ± standard deviations (SD).

Reference

(1) Freshney, D. I. *Culture of Animal Cells: A Manual of Basic Technique*, 5th ed.; Wiley-Liss, 2005.
